# Sometimes It Drains, Sometimes It Sustains: The Dual Role of the Relationship with Students for University Professors

**DOI:** 10.1155/2019/9875090

**Published:** 2019-07-16

**Authors:** Mara Martini, Gloria Guidetti, Sara Viotti, Barbara Loera, Daniela Converso

**Affiliations:** Department of Psychology, University of Turin, Italy

## Abstract

University organizational contexts have been changing significantly in recent years, and academic staff are expected to manage larger workloads at an increased pace. This can threaten their well-being and exacerbate work-related stress—possibly creating negative impacts on their mental and physical states. Surprisingly, academic occupational psychological health is still rarely studied. By referring to the Job Demands-Resources (JD-R) conceptual model, this study aimed to analyze the relationship between university teachers' well-being and job demands and resources, with a particular focus on the role of the relationship with students. Specifically, 550 associate and full professors were studied to determine the impact of job characteristics, quality of relationships in the work environment, and negative and positive relations with students regarding emotional exhaustion and work engagement. Hierarchical multiple regression models allowed us to highlight the fact that emotional exhaustion was positively and significantly associated with workload, conflicts with colleagues, and requests from students, and it was negatively associated with work meaning. Work engagement was positively and significantly associated with work meaning and social support from students. Our study points out that the flexible and renowned JD-R model can successfully be used to analyze the occupational psychological health of academics. Further, our study underscores the fact that, among job demands and resources, the often-neglected relations with external users (the students) can play an important role in university teachers' perceptions of exhaustion and engagement.

## 1. Introduction

Universities in European countries have been experiencing a complex situation in recent years. Research funding has been greatly reduced, but high formative offerings are still expected in a market-oriented approach [1–3]. Within this framework, and considering other possible sources of stress, such as unsatisfying systems of recognition or reward and work-family conflict [4], academics' occupational well-being can be severely threatened. Nevertheless, university professors' well-being can be an important factor in offering quality (formative) services. Several studies [5] show that quality of work is positively related to professionals' levels of well-being.

To analyze university teachers' psychological health, in line with [6], the renowned Job Demands-Resources (JD-R) model [7] can be used. This conceptual model assumes that employees' psychological health can be affected by two main factors: job demands and job resources. Job demands are all aspects of the job that require the worker to exert physical and/or psychological effort, while job resources are those aspects of the job that help achieve work goals and reduce job demands. If job demands are associated with physiological and/or psychological costs, job resources can relieve these costs, thus creating positive occupational well-being outcomes, such as work engagement. Therefore, a dual process induced by both requests and resources takes place. As a result of the first process (impairment), workers can experience progressive exhaustion due to excessive demands. In the second process (motivational), employees can improve their ability to cope with demands if resources are available. As a result, they can increase motivation, satisfaction, and work engagement. Many studies [8] within the conceptual JD-R model framework consider requests and resources referring to the work content relationship quality inside the organization. Among job requests, one of the most relevant is the workload based on the quantity of tasks requiring completion and the pressure to finish them [7]. Conversely, based on performance feedback or activity meaning or significance (frequently) [9], the work itself can offer sustenance that can enhance motivation. Other important sources of requests and resources at work are at the interpersonal level. Conflicts in work relationships can indeed be emotionally demanding for workers [10]. However, positive and supportive relationships with supervisors and coworkers are among the most cited [6, 10–14] resources that can sustain the motivation process, thus reducing burnout risk and enhancing work engagement.

One of the main strengths of the JD-R model is its ductility, as it can include both general and specific demands and resources for all kinds of professions and organizational contexts. Previous studies [6] demonstrated that particular sources of stress for academics are the workload in terms of bureaucratic requirement deadlines, an increasing number of students to look after, and conflicting relationships with colleagues (see, among the others, [3, 15]). However, the intrinsic reward or source of meaning, namely, the pedagogical impact of teaching, can be a resource for academics [16]. Notwithstanding, for professions in which the relationship with users represents a central dimension (e.g., education sector, nursing profession, and public services), with few exceptions [16], demands and resources from users have rarely been considered [17]. More precisely, several authors underline the demanding role that can be played by disproportionate requests [18–20], mistreatment [21], or aggressive behaviors [22, 23] from users or by prolonged monodirectional supply of caring [17, 24]. The negative side of relationships with users or clients is largely described as a source of requests that can drain professionals' psychological resources and lead to emotional exhaustion. To counterbalance this relational depletion, a large part of the extant literature considers the buffering role played by social support in containing stress and burnout [25, 26]. Traditionally, the most cited sources of social support at work are, as previously described, within the organization, colleagues, supervisors, and/or the organization itself [6, 11–13]. Although less considered as a source of support, the positive relationships with users or clients, proposed by Zimmermann et al. [27] for retail sector workers and then developed by Loera et al. [28] for educational professionals, play an important role in enhancing workers' professional accomplishment. Dealing with people is one of the main reasons reported by some workers in the educational and health sectors for having chosen their profession. In different kinds of people-oriented professions [17, 27–29], workers are offered an indirect reciprocity in terms of meaning in their work and a direct reciprocity in terms of the gratitude and social support expressed by users.

As Zimmermann et al. [27] clearly describe, support from users or clients can perform different functions: instrumental, emotional, cognitive, or informative. Support serves an instrumental function when it helps to solve practical problems through concrete actions. It serves an emotional function if it offers feelings of comfort, caring, or safety or leads people to believe they are admired and valued. Support serves a cognitive or informative function if it provides knowledge and tips that help people face issues and understand their context. Due to the specific nature of university teacher-student relationships, students can hardly offer instrumental support to teachers. However, students can offer academics forms of emotional support through expressions of recognition and gratitude. They can also sometimes provide cognitive or informative support by making useful information available or by simply avoiding overwhelming teachers with unnecessary or excessive emails or requests. A form of both cognitive and emotional support specific to the teacher-student relationship can then be the evidence of the students' learning and intellectual growth. Indeed, it can represent a source of motivation for teachers, as reported by Darabi et al. [30] and theorized by Hagenauer and Volet [31]. These authors underline the importance of increasing the study of reciprocity in teacher-student relationships in terms of affective and supportive dimensions. In their analyses, they affirm that quality of teaching (by teachers) and learning (by students) can become a virtuous circle only if the two directions of the positive relationship are considered and enhanced. If this circle is achieved, positive satisfaction-related outcomes are likely for all actors.

Therefore, in the theoretical framework of the JD-R model [25], the aim of the present research was to study academics' psychological health by analyzing how teachers' perceptions of emotional exhaustion (impairment process) and work engagement (motivational process) are related to both “classical” demands and resources (content of work and quality of relationships in academia) and the demands and the resources represented by relations with users of the university (the students). More specifically, our hypotheses stated the following.

- H1: Requests referring to content of work (work overload) and to relationships in academia (conflict) are positively related to emotional exhaustion (H1a) and negatively related to work engagement (H1b).
- H2: Resources referring to content of work (meaningfulness of work) and to relationships in academia (social support from colleagues) are positively related to work engagement (H2a) and negatively related to emotional exhaustion (H2b).
- H3: Students' excessive demands are positively related to emotional exhaustion (H3a) and negatively related to work engagement (H3b). When considering the relationship with students, the quality of the regression model solution increases (H3c).
- H4: Students' support is positively related to work engagement (H4a) and negatively related to emotional exhaustion (H4b). When considering the relationship with students, the quality of the regression model solution increases (H4c).
- H5: In line with previous studies [8], since the two separate impairment and motivational processes are determined by the roles of job demands and resources, the demands are more strongly related to emotional exhaustion than the resources (H5a), and the resources are more strongly related to work engagement than the demands (H5b).

## 2. Materials and Methods

### 2.1. Procedure and Participants

During 2017, data were collected in several departments of a large university in northern Italy. An online survey was developed and proposed to teaching and research staff. The questionnaire was composed of a sociodemographic section and scales to evaluate job demands, job resources, and occupational well-being outcomes.

In total, 1012 questionnaires were completed (52% of the entire population). Of these, 550 (54.3 percent of the returned questionnaires) were considered for analysis. The inclusion criterion included fulfilling a minimum 120-hour teaching load for the year. For these academics, their relationships with students are a significant component of their jobs. Moreover, questionnaires with at least one missing piece of data regarding the single items of the considered scales were excluded from the final sample. Therefore, the final sample was composed of the responses of 369 (67.1 percent) associate professors (APs) and 181 (32.9 percent) full professors (FPs). Regarding gender composition, 309 were male (56.2 percent) and 241 were female (43.8 percent). Their mean age was 53 years (SD = 7.96).

The entire population was fully informed about the research aims and outcomes. The participants volunteered for the research without receiving any reward, and they agreed to complete the questionnaire anonymously. The research conforms to the Declaration of Helsinki of 1995 (as revised in Edinburgh in 2000), and all required ethical guidelines for conducting human research were followed, including adherence to the legal requirements of the country under study. No treatment, including medical, invasive diagnostics, or procedures causing psychological or social discomfort, was administered to the participants; therefore, no additional ethical approval was required.

### 2.2. Measures

To evaluate job characteristics, we considered the perception of workload as a job demand and the perception of work as a job resource for university teachers.

- Work overload (WO) was measured using seven items adapted from Melin et al. [32] (e.g.,* I work with many different work tasks at the same time.*). The scale ranged from 0 (totally disagree) to 3 (totally agree).
- Meaningfulness of work (MW) was measured using five items from the Copenhagen Psychosocial Questionnaire [33] (e.g.,* I think that my work is meaningful.*). The responses were given on a four-point scale ranging from 0 (totally disagree) to 3 (totally agree).

To analyze the quality of relationships in the work environment, we considered the negative dimension of conflict among colleagues and the positive dimension of perceived social support from colleagues.

- Conflict (CO) was measured using four items from the Multidimensional Organizational Health Questionnaire [34] (e.g.,* there are people who are marginalized.*). The scale ranged from 0 (totally disagree) to 3 (totally agree).
- Social support from colleagues (SSCo) was measured using four items from the Italian version [35] of the Health and Safety Executive (HSE) Work-Related Stress Indicator Tool [36] (e.g.,* I receive the help and support I need from my colleagues.*). The answer scale ranged from 0 (totally disagree) to 3 (totally agree).

Relationships with students were examined by considering both the positive and the negative sides of the relationships. The negative side was examined based on excessive demands posed by the students, which represent a stressful aspect of the relationship. Conversely, the positive side reflects the dimension of support experienced in the relationship with the students. More precisely, the scale evaluates whether academics feel that their work is appreciated and recognized by the students.

- Students' excessive demands were measured using four items adapted from the excessive customer expectations dimension of the Customer-Related Social Stressors (CSS) scale [9] (e.g.,* students make excessive demands.*).
- Students' support was measured using four items adapted from the User-Initiated Support Scale (UISS) [28] (e.g.,* students explicitly appreciate my way of working.*).

The responses of both scales were given on a four-point scale ranging from 0 (not at all) to 3 (always).

Occupational psychological health was analyzed using the two dimensions that are the results of the impairment and motivational processes described by the JD-R model [7], respectively.

- Emotional exhaustion (EE) was measured using the corresponding five-item subscale from the Maslach Burnout Inventory–General Survey (e.g.,* I feel emotionally drained by my work.*) [37].
- Work engagement (WE) was measured using the nine-item Italian version of the Utrecht Work Engagement Scale (UWES) [38, 39], which was considered a one-dimensional scale (e.g.,* at work, I feel that I am bursting with energy.*).

Responses to the burnout and work engagement measures were provided on a scale ranging from 0 (never) to 6 (every day).

## 3. Statistical Analysis

Data analyses were performed using the IBM SPSS, Version 25, statistics package for Windows. For each scale of the questionnaire, internal consistency was assessed by the Cronbach's alpha coefficient, and synthetic indexes were then calculated. After descriptive (mean [M] and standard deviation [SD]) analysis of each synthetic index, hierarchical multiple regression models were performed to evaluate which demands and resources influenced academics' psychological health at work. We specified two separate regression models for emotional exhaustion and work engagement, the two dependent variables. In the hierarchical regression process, predictor variables are added in successive steps (enter method) based on their theoretical status. In the first step, we inserted demographic control variables (e.g., gender, age, and academic role). To control for the covariation between EE and WE, EE was entered at the first step when WE was the dependent variable, and WE was entered at the first step when EE was the dependent variable. In the second step, we added a demand and a resource referring to work content: negative and positive dimensions of relationships within the university context. These represent relationship conflicts among colleagues and social support from colleagues. Finally, in step three, we inserted the negative and positive sides of relationships with external users of the university: exceeding excessive demands (CSS) and social support (UISS) from students. This model estimation process allowed us to evaluate if, after adding new predictive variables, the predictors inserted in the later steps explained a significant portion of the variance over and above the variables inserted at the previous steps. Then, at each step, the holistic fit index useful for evaluating the model's solution quality (R^2^ coefficient) can increase (ΔR^2^), showing the marginal utility of the most recently added variables.

## 4. Results

Table 1 shows the results of the correlation analysis between all variables considered for the present study, followed by the means, standard deviations, and Cronbach's alphas. The scales and subscales had adequate internal consistency, and all the variables correlated in the expected direction.

Tables 2 and 3 show the results of the multiple regression analysis. At step one, only the control variables were inserted, namely, age, gender, academic role, EE, and WE, respectively, to control for the covariation between them. Academic role was dummy coded, and the reference category was FPs. Age was not significantly associated with EE, whereas with WE, it was significant only in the first step. Moreover, females were more exhausted and more engaged than men. Regarding academic position, no significant association emerged regarding EE, whereas for WE, it stopped being significant after the first step. Moreover, WE was negatively and significantly associated with EE (Table 3), and EE was significantly and negatively associated with WE (Table 3).

In step two, job characteristics and variables regarding employees' relationships in the work environment were entered. WO had a significant and positive relationship with EE only, while MW showed a negative relationship with EE and a positive relationship with WE.

Moreover, relationship conflict among colleagues (CO) was positively and significantly associated with EE only, whereas perception of social support from colleagues (SSCo) was significantly and positively associated with WE only (Table 3). The change in R^2^ was significant for both the regression models with EE and the model with WE as a dependent variable, increasing when adding the step two variables above to control variables.

Finally, in step three, we entered the relationship with students' variable in its positive and negative aspects, namely, the excessive demands posed by students and the students' support. Specifically, as shown in Tables 2 and 3, CSS had a significant and positive influence on EE only, while UISS had a significant and positive impact on WE only. As the students' excessive demands increased, EE increased, and as students' support increased, WE increased. After considering positive relations with students (UISS), it is noticeable that social support from colleagues (SSCo) showed no further significant impacts on WE in the last step (Table 3). The change in R^2^ was significant for both regression models. When adding variables concerning the relationship with students, the increase in R^2^ was quite strong for the model with EE as the dependent variable; however, it was less intense for the model with WE as the dependent variable.

## 5. Discussion and Conclusions

Within the conceptual framework of the JD-R model [7], the aim of the present study was to analyze the role of the quality of relationships with external users, namely, the perceived excessive requests or supportive behavior by students, over and beyond the role of some job demands and resources characteristics. Among these we considered work overload and work meaning, and the negative and positive side of the social work environment, such as conflict relationships with colleagues and support from colleagues.

Results of the hierarchical regression models allowed us to partially confirm our initial hypotheses. Specifically, H1a was fully confirmed, as requests regarding content of work (work overload) and relationships in academia (conflict) were positively related to emotional exhaustion. However, H1b was not proved because the data did not show any significant relationship between work overload or conflict and work engagement at step 2 of the regression model.

H2a was partially confirmed, as meaningfulness of work was related to work engagement, but social support from colleagues was not significantly related to work engagement at step 3 of the regression model. Similarly, H2b was partly verified, as only meaningfulness of work was negatively related to emotional exhaustion.

Moreover, for the academics, the study showed that the relationship with students can represent both a demand, which is associated with higher levels of emotional exhaustion, and a resource, when students are supportive. Student support is indeed positively associated with higher levels of work engagement.

The two hypotheses concerning the relationship with students were thus partially confirmed. H3a and H4a, respectively, were verified, as students' excessive demands were positively related to emotional exhaustion and students' support was positively related to work engagement. However, H3b and H4b were not confirmed: students' excessive demands were not related to work engagement, and students' support did not relate to emotional exhaustion. Notably, regarding work engagement, the significant relationship with support from colleagues disappeared when inserting supportive relationships with students. Also, H3c and H4c found confirmation: the results showed a weak, but significant, increase in the fit of the regression models after the introduction of requests and resources from the relationship with students. The results indicate that students' recognition of teachers' commitment to work and appreciation of the way they work represent a valued resource that could sustain the motivational outcome of work engagement. This is in line with the results of Hamilton's [14] qualitative study.

Moreover, even if EE and WE were related, as shown by the two regression models, the results were in line with previous studies where they represented two independent constructs [39] that were the results of two independent processes. This confirms H5. Indeed, work overload, conflict, and students' demands were strongly related to emotional exhaustion, whereas only the meaningfulness of work resource had a significant negative role in the impairment process. However, the meaningfulness of work and students' support resources were strongly related to work engagement.

Considering control variables, gender played an influential role in both negative and positive aspects of psychological health at work. This suggests that, for women, the perceptions of both emotional exhaustion and work engagement were higher. Regarding the academic role, full professors were significantly more engaged than associate professors, even if this relationship disappeared when the job demands and resources roles were assessed.

Overall, the results of the present study were consistent with Mudrak and colleagues' study [6]. This research was conducted in the Czech Republic, and it analyzed academics' occupational well-being. After the changes that have impacted university systems, such as funding reduction and workload increases for academics who have to simultaneously manage teaching, research, and bureaucratic and fundraising activities, academics' psychological health has indeed become a topic of attention. Nevertheless, until now, academics' occupational well-being has not been widely analyzed in terms of the specific demands and resources that characterize their work environment. Therefore, our work offers a further contribution to deepening and refining the focus on a cultural context that, compared with the Anglo-American one in which most of the studies have been conducted, is still underrepresented in this field of research.

Considering occupational psychological health in the conceptual frame of the JD-R model [7, 25], only demands and resources related to job content and relations inside the organization are traditionally considered—even for professionals and academics [6] who spend a considerable part of their activities relating with users. External users are sometimes considered sources of requests [18, 24] but are much more rarely considered sources of social support [27, 28]. Moreover, the present study showed the relevance of the positive side of the academic-student relationship. In a historical period that poses numerous requests and challenges to academics, it should be underlined how the relationship with students represents an important source of support, which, according to previous studies, is the core of the teaching role [30].

Finally, this study is not without limitations. First, it involved only academics from an Italian university, so results cannot be generalized. Future works could involve participants at universities in several regions of Italy and possibly other countries. Second, future studies should also consider a wider plethora of job demands and resources that are specific to the academic context, such as work-family conflict or the role of the multiple academic tasks related to teaching, research, and third-stream activities. Moreover, the study is cross-sectional, so the direction of the explored relationships could not be verified. In the future, we can utilize repeated administrations of the instrument to analyze these results over time.

Despite these limitations, the present study is valuable, as it offers an analysis of the still underexamined topic of academics' psychological health at work using a renewed and ductile conceptual frame, the JD-R model, in line with Mudrak et al.'s work [6]. This research design can also be adopted in other countries to study demanding and supportive aspects of the university environment to improve occupational well-being for academics.

## Figures and Tables

**Table 1:** Descriptive statistics and bivariate correlations.

	1	2	3	4	5	6	7	8	9	10	11
1 Age	1	-.053	.467*∗∗*	-.154*∗∗*	-.029	-.057	-.014	-.133*∗∗*	.074	-.020	-.093*∗*
2 Female		1	-.119*∗∗*	.030	-.041	.021	-.065	.033	-.041	.048	.127*∗∗*
3 FP			1	.036	.132*∗∗*	-.062	.140*∗∗*	-.036	.068	.114*∗∗*	-.114*∗∗*
4 WO				1	-.015	.198*∗∗*	-.110*∗∗*	.226*∗∗*	-.124*∗∗*	-.037	.415*∗∗*
5 MW					1	-.172*∗∗*	.299*∗∗*	-.210*∗∗*	.348*∗∗*	.577*∗∗*	-.307*∗∗*
6 CO						1	-.390*∗∗*	.203*∗∗*	-.113*∗∗*	-.190*∗∗*	.295*∗∗*
7 SSPo							1	-.107*∗*	.254*∗∗*	.279*∗∗*	-.244*∗∗*
8 CSS								1	-.454*∗∗*	-.123*∗∗*	.294*∗∗*
9 UISS									1	.291*∗∗*	-.226*∗∗*
10 WE										1	-.271*∗∗*
11 EE											1
*M*				12.77	11.72	3.46	6.09	2.71	6.79	40.04	11.34
*SD*				3.79	2.63	2.68	2.59	2.01	2.22	9.03	7.07
*Alpha*				.79	.85	.84	.84	.76	.77	.89	.85

*∗*p < .05; *∗∗* p<.01. Gender: 0, M; 1, F. Work role: AP (associate professor), 0; FP (full professor), 1; WO, work overload; MW, meaningfulness of work; CO, conflict; SSCo, social support form colleagues; CSS, students' demands; UISS, students' support.

**Table 2:** Regression parameters: standardized coefficients and overall changes in R2 for emotional exhaustion.

	Step 1	Step 2	Step 3
Age	-.076	.003	.020
Female	.133*∗∗*	.098*∗*	.096*∗*
FP	-.031	-.066	-.073
WE	-.275*∗∗∗*	-.105*∗*	-.107*∗*
WO		.191*∗∗∗*	.350*∗∗∗*
MW		-.245*∗∗∗*	-.164*∗∗∗*
CO		.145*∗∗∗*	.127*∗∗∗*
SSCo		-.039	-.039
CSS			.134*∗∗*
UISS			-.001

Adj. R^2^	.095	.304	.318
Δ R^2^	.102*∗∗*	.212*∗∗∗*	.016*∗∗*
R^2^	.102	.315	.331

Method: enter.

*∗*p < .05; *∗∗* p<.01; *∗∗∗* p<.001. Gender: 0, M; 1, F. Work role: AP (associate professor), 0; FP (full professor), 1; WE, work engagement; WO, work overload; MW, meaningfulness of work; CO, conflict; SSCo, social support form colleagues; CSS, students' demands; UISS, students' support.

**Table 3:** Regression parameters: standardized coefficients and overall changes in R2 for work engagement.

	Step 1	Step 2	Step 3
Age	-.108*∗*	-.029	-.030
Female	.095	.097*∗∗*	.098*∗∗*
FP	.145*∗∗*	.050	.050
EE	-.277*∗∗∗*	-.098*∗*	-.100*∗*
WO		-.025	.024
MW		.512*∗∗∗*	.490*∗∗∗*
CO		-.043	-.056
SSCo		.091*∗*	.072
CSS			.068
UISS			.112*∗∗*

Adj. R^2^	.091	.355	.362
Δ R^2^	.98*∗∗∗*	.267*∗∗∗*	.009*∗*
R^2^	.098	.365	.375

Method: enter.

*∗*p < .05; *∗∗* p<.01; *∗∗∗* p<.001. Gender: 0, M; 1, F. Work role: AP (associate professor), 0; FP (full professor), 1; WE, work engagement; WO, work overload; MW, meaningfulness of work; CO, conflict; SSCo, social support form colleagues; CSS, students' demands; UISS, students' support.

## Data Availability

Datasets supporting the conclusions of this article are available and can be requested from the corresponding author.
